# Supplementary material for: Effects of Simulated Nitrogen Deposition and Micro-Environment on the Functional Traits of Two Rare and Endangered Fern Species in a Subtropical Forest
Source: Plants (Basel). 2022 Dec 1;11(23):3320. doi: 10.3390/plants11233320 (PMC9740810; doi:10.3390/plants11233320)
Supplement: Supplementary file 1 [file plants-11-03320-s001.zip › plants-1990134-supplementary.pdf]

Supplementary Material

Results of correlation analysis between traits and between soil properties

For the correlations between traits, the three growth traits of *C. baromet* - SLA, LNC and LPC were positively correlated between each other, and each of them also showed negative correlation with the defense trait of LDMC (Figure S2). Meanwhile, negative relationships between growth traits and defense traits or reproductive traits could also be found. In contrast, there was no correlation between *A. podophylla* growth traits, but negative correlations between its defense traits and positive relations between its reproductive traits were often found (Figure S3). Meanwhile, growth traits often have significant relationships with defense traits, with both positive or negative relationships could be detected depending on the specific traits.

Positive correlations were often found between soil properties (Figure S4). Yet, soil pH frequently showed negative correlations with the other soil properties (except for its strongly positive relationship with NO_3^--N). Both soil C and N positively correlated with soil P, AP, NH_4^+-N and AK.

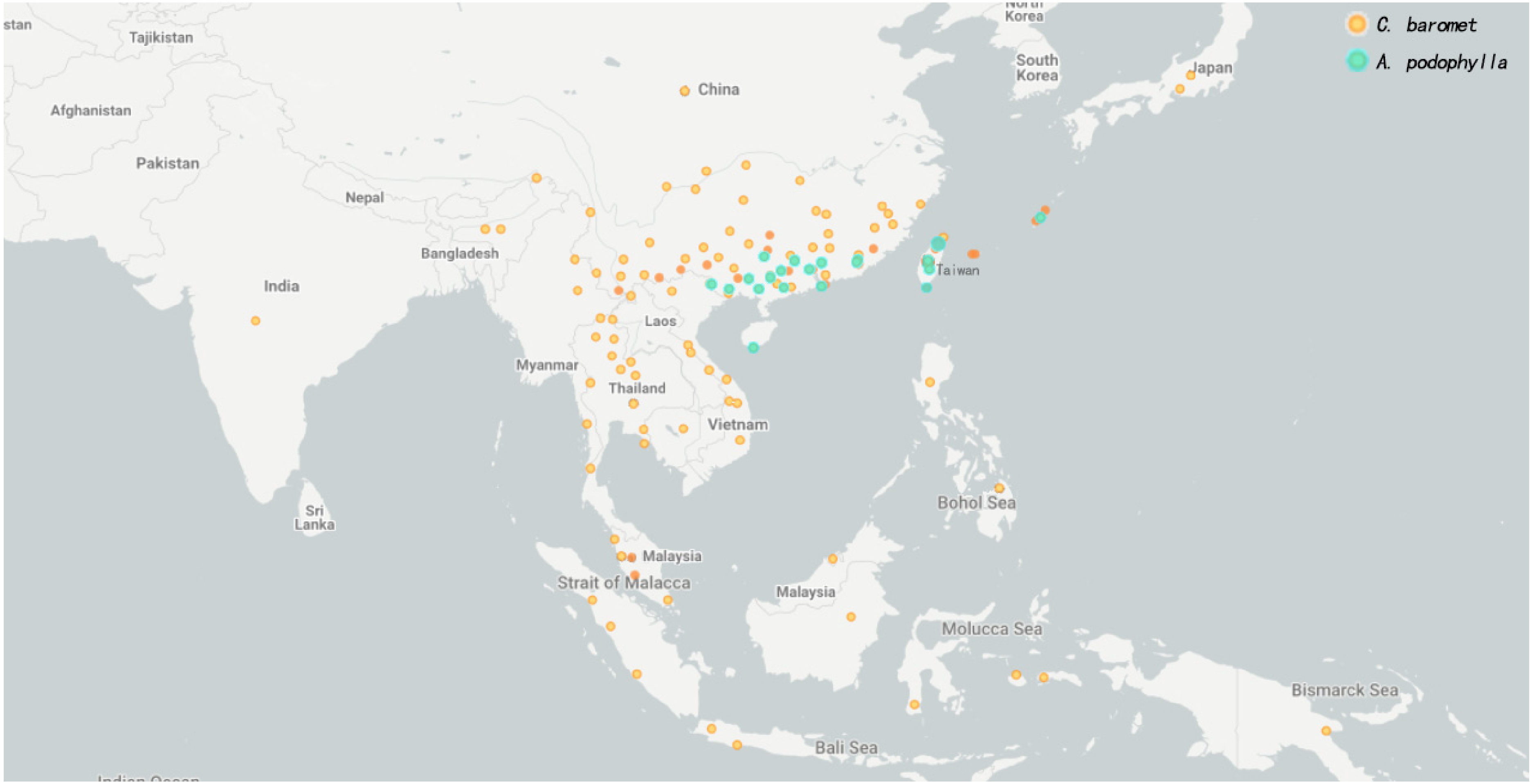

Figure S1. The occurrence coordinates in Asia of the two studied REFs.

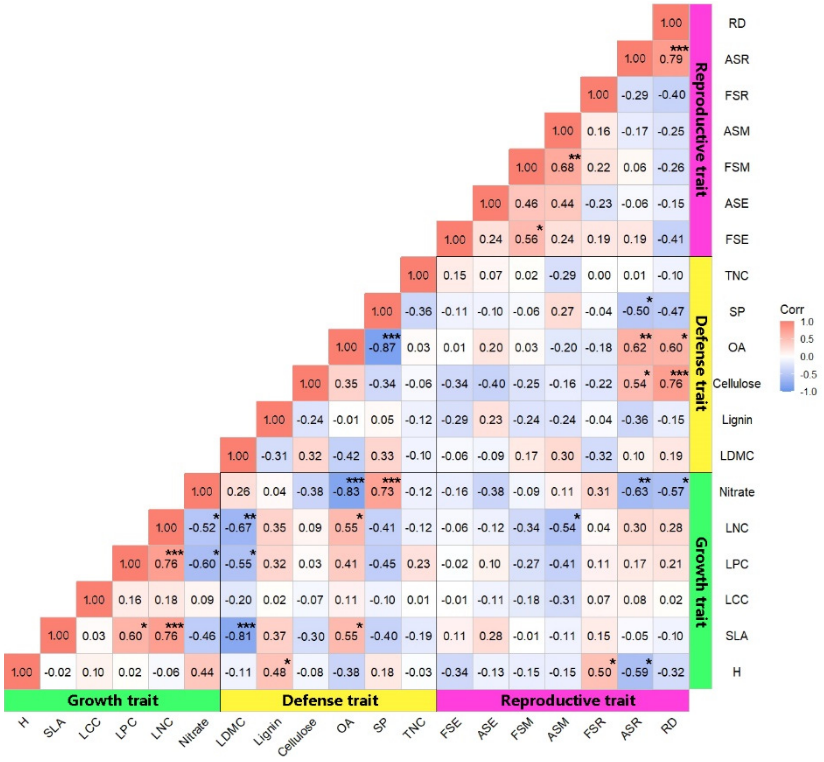

Figure S2. Correlations between traits of *C. baromet*. Abbreviations for the traits are defined in Table 3. "*", "**" and "***" indicate statistical significance at $p < 0.05$, 0.01 and 0.001 respectively.

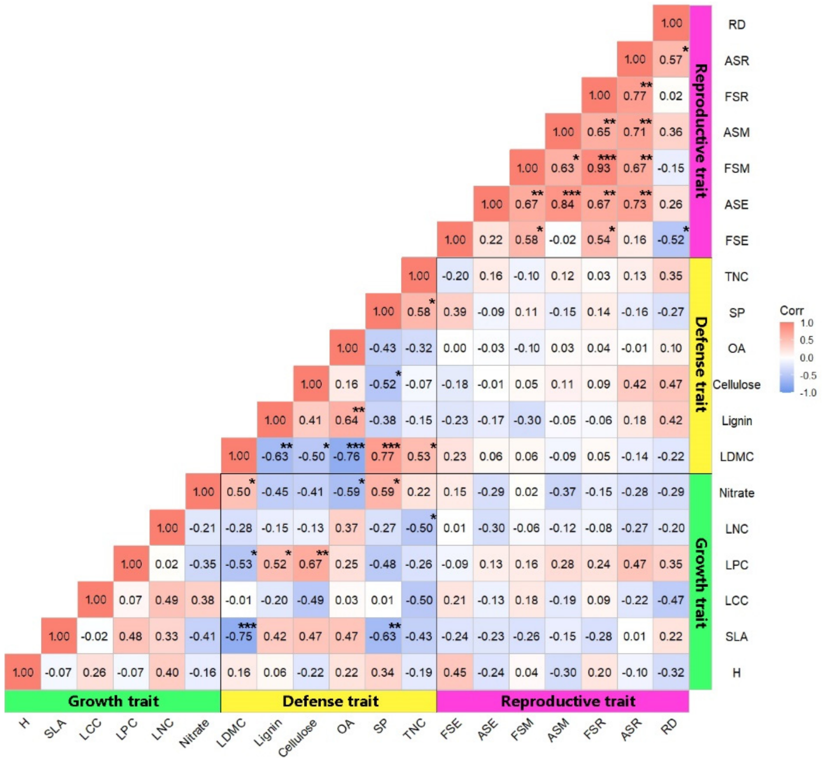

Figure S3. Correlations between traits *of A. podophylla*. Abbreviations for the traits are defined in Table 3. "*", "**" and "***" indicate statistical significance at $p < 0.05$, 0.01 and 0.001 respectively.

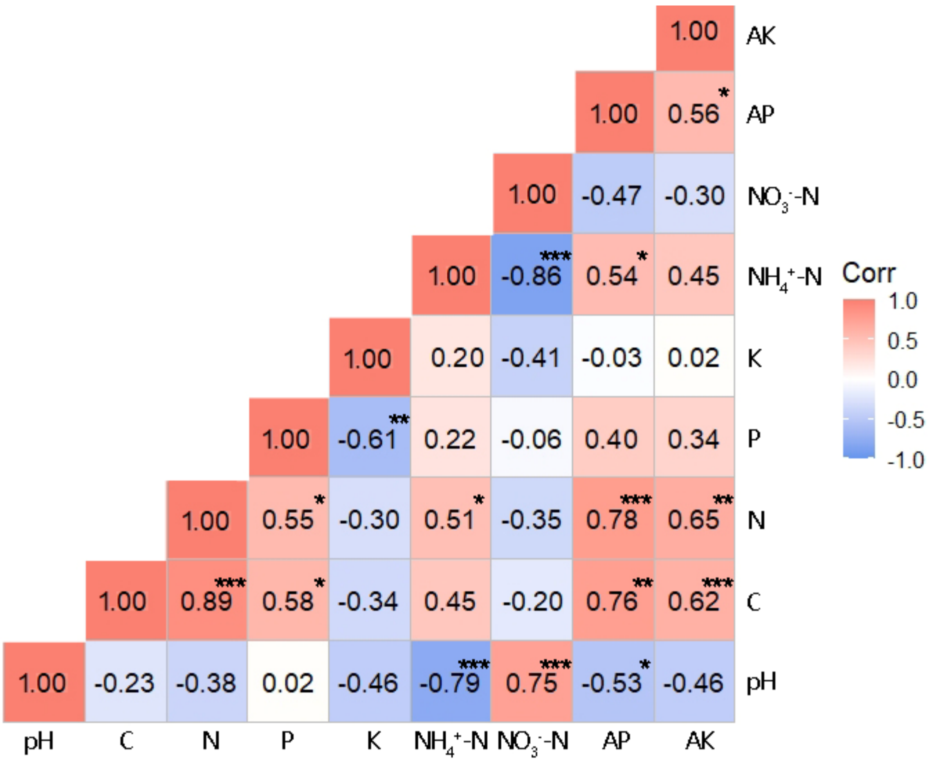

Figure S4. Correlations between soil chemical properties. Abbreviations for the ecological variables are defined in Table 4. "*", "**" and "***" indicate statistical significance at $p < 0.05$, 0.01 and 0.001 respectively.

Table S1. Comparisons of micro-environmental factors among different treatments.

Treatment	pH	C (mg/g)	N (mg/g)	P (mg/g)	K (mg/g)	NH_4^+-N (μg/g)	NO_3^--N (μg/g)	AP (μg/g)	AK (μg/g)	Subcanopy cover (%)
Control	3.71[a]	36.72[a]	2.54[a]	0.32[a]	20.79[a]	4.44[a]	19.72[a]	0.81[a]	33.80[a]	59.63[a]
CAN25	3.72[a]	36.15[a]	2.41[a]	0.34[a]	16.88[a]	3.93[a]	24.33[a]	0.74[a]	38.03[a]	39.99[a]
CAN50	3.68[a]	38.00[a]	2.34[a]	0.32[a]	19.72[a]	3.56[a]	19.22[a]	0.81[a]	33.36[a]	63.37[a]
UAN25	3.68[a]	39.46[a]	2.50[a]	0.32[a]	18.71[a]	4.46[a]	21.06[a]	1.22[a]	35.99[a]	51.72[a]
UAN50	3.66[a]	41.34[a]	2.63[a]	0.32[a]	20.96[a]	8.97[a]	28.68[a]	0.96[a]	37.20[a]	77.26[a]

Abbreviations for the ecological variables are defined in Table 4. The same superscript letters (a) indicate insignificant differences at level $p < 0.05$ among Treatments (Tukey HSD test).

Table S2. The AICc values of each soil model explaining the functional traits of the two studied REFs.

Species	Variable	Growth trait						Defense trait						Reproductive trait						
		H	SLA	LCC	LNC	LPC	Nitrate	LDMC	Lignin	Cellulose	OA	SP	TNC	FSE	ASE	FSM	ASM	FSR	ASR	RD
C. baromet	pH	824.92	230.38	218.72	109.21	45.88	57.96	225.39	99.01	106.16	159.90	296.03	265.04	232.95	233.99	**203.05**	253.85	**200.81**	227.92	232.55
	C	821.35	228.57	219.83	108.73	44.16	**57.52**	224.29	99.02	106.67	159.07	295.95	263.97	235.47	233.94	213.00	255.88	203.21	227.87	233.59
	N	815.46	227.82	219.69	108.98	44.68	58.03	222.52	98.86	105.21	159.91	295.83	264.14	235.38	233.06	212.66	255.64	203.64	226.58	233.23
	P	**815.11**	229.42	223.18	108.93	**41.96**	57.95	224.40	**98.70**	**104.40**	**158.73**	**295.48**	264.62	235.42	233.96	212.61	255.75	201.50	**222.99**	**231.30**
	K	1067.29	234.21	218.83	117.65	45.66	57.86	225.63	124.03	106.48	175.35	313.19	264.70	235.32	**229.89**	207.44	**253.24**	202.74	226.34	232.47
	NH_4^+N	825.93	228.90	222.27	108.68	45.81	57.74	225.51	99.07	106.14	159.27	296.07	265.14	233.97	233.90	211.14	255.78	203.60	227.26	232.95
	NO_3^-N	821.02	230.44	221.34	108.65	45.70	57.98	223.73	98.99	106.71	159.88	296.07	265.12	**229.98**	234.01	207.95	255.84	203.46	227.59	232.58
	AP	822.35	229.40	**217.61**	**107.83**	44.83	58.03	224.57	99.03	106.02	159.52	295.98	**262.15**	234.59	234.03	208.05	253.26	203.49	227.80	232.40
	AK	825.61	**227.12**	223.88	108.89	45.71	57.53	**222.46**	98.74	106.62	159.41	295.88	263.19	235.45	234.04	211.29	255.89	203.29	227.84	233.59
A. podophylla	pH	1068.24	233.10	**241.48**	118.08	50.53	65.43	240.95	128.39	106.34	175.63	312.07	277.97	402.79	307.88	293.41	295.74	266.41	293.32	281.06
	C	1066.01	230.35	247.07	117.71	48.30	65.37	240.22	128.36	**101.94**	175.39	314.47	279.59	**401.09**	304.80	**290.41**	293.96	268.56	292.97	281.28
	N	1068.08	232.25	246.82	116.68	48.58	65.40	241.24	128.38	103.83	175.77	314.71	281.12	402.01	305.70	291.71	294.81	268.37	292.88	281.66
	P	1066.71	231.06	246.99	117.99	48.66	65.44	242.75	128.62	105.90	175.88	315.33	281.36	402.10	305.89	293.99	296.28	267.75	292.26	281.64
	K	1067.29	234.21	246.84	117.65	51.08	64.88	242.98	**126.15**	106.48	175.35	313.19	278.46	403.46	308.19	295.68	300.93	269.35	293.32	281.66
	NH_4^+N	**1054.93**	233.95	243.91	117.77	50.88	65.39	241.58	128.78	106.48	175.75	314.36	280.94	401.85	306.65	293.28	294.29	268.09	291.59	281.29
	NO_3^-N	1060.06	233.43	244.24	117.43	50.94	64.91	240.02	127.94	105.57	175.60	315.32	279.85	402.67	307.81	292.94	294.96	265.51	293.27	281.60
	AP	1068.26	**229.97**	246.03	118.08	**47.49**	64.61	**234.49**	126.94	104.18	**173.34**	**310.18**	**275.87**	401.54	306.91	292.92	295.57	268.66	293.31	281.31
	AK	1067.82	234.36	246.58	**114.81**	51.09	**63.55**	243.13	128.79	106.58	175.52	315.23	280.98	403.10	**304.22**	291.56	**293.04**	**264.71**	**291.29**	**280.95**

Abbreviations for the traits and ecological variables are defined in Table 3 and Table 4, respectively. For each trait, the model with the lowest AICc value is in bold.

Table S3. Effects of the best models related to micro-environment on the growth traits of the two studied REFs.

Species	Model	Variable	H			SLA			LCC			LNC			LPC			Nitrate		
			Estimate	SE	*P*	Estimate	SE	*P*	Estimate	SE	*P*	Estimate	SE	*P*	Estimate	SE	*P*	Estimate	SE	*P*
C. baromet	P	Intercept	90.34	47.25	0.06										-0.24	0.90	0.79			
		P	181.4	164.28	0.27										5.82	2.81	**0.05**			
	NH_4^+N	Intercept																1.59	0.19	<0.01
		NH_4^+N																0.05	0.03	0.61
	AK	Intercept				139.15	38.76	<0.01												
		AK				1.93	1.07	0.08												
	Subcanopy cover	Intercept										23.90	0.85	<0.01						
		Subcanopy cover										0.02	0.01	0.11						
A. podophylla	pH	Intercept							932.38	197.72	<0.01									
		Soil pH							-130.31	53.79	**0.02**									
	AP	Intercept				201.87	9.4	<0.01							1.09	0.24	<0.01			
		Soil AP				20.26	9.64	**0.04**							0.47	0.25	0.07			
	AK	Intercept										31.97	3.82	<0.01						
		Soil AK										-0.19	0.11	0.09						

Abbreviations for the traits and ecological variables are defined in Table 3 and Table 4, respectively. Bold values indicate statistical significance at $p < 0.05$.

Table S4. Effects of the best models related to micro-environment on the defense traits of the two studied REFs.

Species	Model	Variable	LDMC			Lignin			Cellulose			SP			TNC		
			Estimate	SE	*P*	Estimate	SE	*P*	Estimate	SE	*P*	Estimate	SE	*P*	Estimate	SE	*P*
C. baromet	P	Intercept				18.23	2.76	<0.01									
		P				5.73	8.62	0.51									
	AK	Intercept	461.44	36.14	<0.01												
		AK	-1.93	0.99	0.06												
	Subcanopy cover	Intercept				20.52	0.69	<0.01									
		Subcanopy cover				-0.01	0.01	0.45									
A. podophylla	C	Intercept							25.92	2.1	<0.01						
		C							0.12	0.06	**0.04**						
	AP	Intercept	327.45	10.19	<0.01							360.34	46.92	<0.01	341.53	24.31	<0.01
		AP	-31.17	10.44	**<0.01**							-109.79	48.08	**0.03**	-64.10	24.91	**0.02**

Abbreviations for the traits and ecological variables are defined in Table 3 and Table 4, respectively. Bold values indicate statistical significance at $p < 0.05$.

Table S5. Effects of the best models related to micro-environment on the reproductive traits of the two studied REFs.

Species	Model	Variable	FSE			ASE			FSM			ASM			FSR			ASR			RD		
			Estimate	SE	*P*	Estimate	SE	*P*	Estimate	SE	*P*	Estimate	SE	*P*	Estimate	SE	*P*	Estimate	SE	*P*	Estimate	SE	*P*
C. baromet	pH	Intercept							-10.23	59.37	0.86				153.57	66.57	0.03						
		pH							53.9	16.11	**<0.01**				32.10	18.05	0.09						
	K	Intercept				177.51	10.85	<0.01				268.44	23.61	<0.01									
		K				-1.06	0.52	**0.04**				-1.8	1.12	0.12									
	NH_4^+N	Intercept																					
		NH_4^+N																					
	NO_3^-N	Intercept	121.98	6.93	<0.01																160.7	20.41	<0.01
		NO_3^-N	-0.71	0.3	**0.02**																0.03	0.1	0.92
	AP	Intercept																329.52	2.62	<0.01			
		AP																0.53	2.53	0.84			
	Subcanopy cover	Intercept										233.91	6.13	<0.01									
		Subcanopy cover										-0.17	0.09	0.07									
A. podophylla	pH	Intercept																			161.12	77.88	0.05
		pH																			-16.37	21.12	0.44
	C	Intercept	132.04	14.11	<0.01							208.89	18.59	<0.01									
		C	-0.58	0.37	0.12							-0.74	0.48	0.13									
	AK	Intercept													224.84	19.39	<0.01	254.16	30.18	<0.01			
		AK													-1.18	0.54	**0.03**	-1.24	0.84	0.15			

Abbreviations for the traits and ecological variables are defined in Table 3 and Table 4, respectively. Bold values indicate statistical significance at $p < 0.05$.
